# Ventricular arrhythmias not meeting criteria for terminating cardiopulmonary exercise testing stratify prognosis and disease severity in heart failure of preserved, midrange, and reduced ejection fraction

**DOI:** 10.1002/clc.23367

**Published:** 2020-04-09

**Authors:** Dejana Popovic, Ross Arena, Djordje Jakovljevic, Arsen Ristic, Marco Guazzi

**Affiliations:** ^1^ Clinic for Cardiology, University Clinical Center Serbia University of Belgrade Belgrade Serbia; ^2^ Department of Physical Therapy, College of Applied Science University at Illinois Chicago Illinois USA; ^3^ Cardiovascular Research Centre, Institute of Cellular Medicine, Medical School, Newcastle University & Newcastle upon Tyne Hospitals NHS Foundation Trust Newcastle upon Tyne UK; ^4^ Heart Failure Unit and Cardiopulmonary Laboratory, University Cardiology Department, I.R.C.C.S Policlinico San Donato University Hospital Milan Italy

**Keywords:** cardiopulmonary exercise testing, prognosis, HFpEF, HFmrEF, HFrEF

## Abstract

**Background:**

Continued high mortality in heart failure patients indicates the need for additional methods of risk stratification and phenotyping.

**Hypothesis:**

We hypothesized that ventricular arrhythmias that do not meet test‐termination criteria (non‐terminating ventricular arrhythmias [NTVA]) during cardiopulmonary exercise testing (CPET) may help in phenotyping disease severity and prognosis in heart failure with reduced (HFrEF) and midrange (HFmrEF)/preserved (HFpEF) left ventricular ejection fraction (LVEF).

**Methods:**

About 319 patients with heart failure (199 HFrEF; 80 HFmrEF; 41 HFpEF) underwent CPET. Tricuspid annular plane systolic excursion (TAPSE) and pulmonary artery systolic pressure (PASP) were measured by echocardiography. B‐type natriuretic peptide (BNP) at rest and peak exercise was also determined. The patients were tracked for primary (cardiac death) and secondary composite outcomes (all‐cause death, heart transplantation/left ventricular assist device implantation, hospitalization for cardiac reasons).

**Results:**

Forty‐seven (15%) of the patients demonstrated NTVA during CPET, regardless of coronary artery disease prevalence. Patients without arrhythmias had a significantly higher LVEF (*P* < .05), TAPSE/PASP ratio (*P* < .001), peak oxygen consumption (*P* < .01), lower resting and peak BNP (*P* < .001), and the minute ventilation/carbon dioxide production slope (*P* < .001) compared to those with NTVA. Seventy‐one patients died during the tracking period, 54 for cardiac reasons. NTVA during CPET was a significant predictor of primary and secondary outcomes in the total heart failure cohort (HR: 5.3, 3.7; 95% CI: 3.1‐9.1, 2.4‐5.5; *P* < .001, respectively), as well as in subgroups categorized according to reduced and middle‐range/preserved LVEF (*P* < .001).

**Conclusion:**

Exercise‐induced ventricular arrhythmias that do not reach test‐termination criteria are nonetheless indicative of an advanced disease severity phenotype and worse prognosis.

## 1 INTRODUCTION

Ventricular arrhythmias (VA) may cause or be a consequence of heart failure (HF). They are common, increase in frequency according to disease severity and portends poor prognosis.^1^, ^2^ VA may be specifically associated with an ischemic etiology in HF^1^, ^3^; however randomized trials do not show a reduction in overall mortality by revascularization therapies.^4^ There is evidence that more than 10 premature ventricular beats per hour and nonsustained ventricular tachycardia (NSVT) increase mortality risk in patients with structural heart disease, although providing little discrimination between sudden cardiac death or death due to progressive HF.^5^ Some other reports demonstrate that in patients with HF and an ejection fraction (EF) below 35%, premature ventricular beats did not have prognostic value beyond other clinical variables.^6^ Exercise may be associated with VA, indicating a higher risk of all‐cause mortality, sudden cardiac death or acute coronary syndrome.^7^, ^8^, ^9^ In HF patients, cardiopulmonary exercise testing (CPET) is a standard of measure, whose main derived variables also have prognostic independent information for risk of sustained VA in HF.^10^, ^11^ The incidence of exercise‐induced VA in patients with HF is high,^7^ with limited and mixed evidence of its prognostic value. Moreover, a number of HF patients may develop VA during CPET that do not meet test‐termination criteria set by guidelines (ie, sustained ventricular tachycardia).^12^ The data on specific prognostic value of exercise non‐terminating ventricular arrhythmias (NTVA), such as ectopic beats or NSVT, are not conclusive suggesting or excluding an increased risk of death.^7^, ^8^, ^9^ In one report of asymptomatic adults, exercise induced NSVT was reported in nearly 4% and no association with mortality was observed.^13^ Data inconsistency may point toward a lack of prognostic value of NTVA in the general population, as well as for HF.

Given the important clinical implications and current lack of firm evidence in the area, the purpose of the current investigation was to define the prognostic significance of NTVA during CPET in a HF cohort across left ventricular ejection fraction category.

## 2 METHODS

### 2.1 Patients

From 1994 to 2015, 319 consecutive HF patients were referred for a clinically indicated hemodynamic and functional assessment at the Cardiomyopathy Program at the Cardiopulmonary Laboratory at San Paolo Hospital, University of Milano.

Subjects underwent a 2D echocardiographic/Doppler evaluation and CPET. Inclusion criteria were (a) signs and symptoms of HF and (b) adequate echocardiographic windows. The diagnosis of HF was based on the recommended criteria of the European Society of Cardiology.^1^ When left ventricular ejection fraction (LVEF) was ≥50%, along with the additional proposed criteria,^1^ patients were considered to have a HF with preserved EF (HFpEF); when EF was 40‐49% they were classified as midrange EF (HFmrEF), and when EF was <40%, patients were classified as HF with reduced EF (HFrEF). We considered ischemic all patients with documented coronary artery disease (CAD; myocardial infarction, revascularization, ≥ 50% reduction in luminal diameter on coronary angiography). The study was approved by the local Ethical Institutional Review Board and informed consent was obtained from all subjects.

### 2.2 Event tracking and endpoints

Subjects were followed for primary outcome (cardiac death) and secondary outcome of composite cardiac events (all cause death, heart transplantation and left ventricular assist device [LVAD] implantation, rehospitalization for cardiac reasons), via hospital and outpatient medical chart review for up to maximum 193 months. Subjects were followed by the HF program providing a high likelihood that all events were captured. Cardiac death was considered to be death due to cardiac reasons, and hospitalization for cardiac reasons and admission to the heart failure unit.

### 2.3 Echocardiography

A 2D and Doppler echocardiography was performed with a Hewlett‐Packard 77 020/A (Andover, MA) and Philips IE33 devices (Andover) by two experienced cardiologists following current guidelines.^14^ A prespecified protocol was used to optimize RV imaging.^15^ The tricuspid annular plane systolic excursion (TAPSE) was obtained by M‐mode analysis in the apical four‐chamber view and was measured as the total displacement of the tricuspid annulus (millimeters) from end‐diastole to end‐systole, on an average of three to five beats.^15^ Pulmonary artery systolic pressure (PASP) was estimated by Doppler echocardiography from the systolic right ventricular to right atrial pressure gradient using the modified Bernoulli equation. Right atrial pressure (assessed jugular venous pressure), estimated by size and respiratory variation of vena cava inferior, was added to the gradient to yield PASP.^14^, ^15^ TAPSE/PASP ratio, a measure of right ventricular‐pulmonary vasculature (RV‐PV) coupling,^16^ was derived. In cases of HFpEF, care was taken to identify the proper etiology of coexistent pulmonary hypertension excluding idiopathic pulmonary arterial hypertension. Accordingly, we referred to Opotowsky et al^17^ proposed and validated 5‐point prediction score based on the measurements of E/e′, the anteroposterior diameter of the left atrium and notching and/or shortened acceleration time of pulmonary flow.

### 2.4 Blood analysis

Blood for NT‐pro‐BNP analysis (20 mL) was taken at rest and at the peak effort, from intravenous cannula placed into the patient's brachial vein before the test. Samples were kept at −80°C and centrifuged on 4000 Hz. NT‐pro‐BNP was measured in all samples by immunoassay sandwich technique (pro‐BNP II, Cobas, Roche, Burgess Hill, England) with lower sensitivity limit of 5 pg/mL.

### 2.5 Exercise testing procedures

Symptom‐limited CPET was performed on a bicycle ergometer for all subjects, according to established guidelines.^18^ Pharmacologic therapy was maintained during CPET. Ventilatory expired gas analysis was performed using a Sensormedics metabolic cart (Vmax, Yorba Linda, CA). Standard 12‐lead electrocardiograms were obtained after adequate skin preparation, at rest, each minute during exercise, and for at least 5 minutes during the recovery phase. Heart rate (HR) was determined at rest, peak exercise and after 1 minute of recovery (HHR‐1). Minute ventilation (VE, BTPS), oxygen uptake (VO_2_, STPD), and carbon dioxide output (VCO_2_, STPD) were acquired breath‐by‐breath and printed using rolling averages every 10 seconds. Peak VO_2_ and peak respiratory exchange ratio (RER) were expressed as the highest 10‐second averaged sample obtained during the last 20 seconds of testing. VE and VCO_2_ values, acquired from the initiation of exercise to peak, were input into spreadsheet software (Microsoft Excel, Microsoft Corp., Bellevue, WA) to calculate the VE/VCO_2_ slope via least squares linear regression (*y* = *mx* + *b*, *m* = slope). Exercise oscillatory ventilation during CPET was defined as previously described in detail.^18^ Test termination criteria consisted of symptoms (ie, dyspnea and/or fatigue), sustained ventricular tachycardia (VT) and NSVT that interfered with hemodynamic stability, > 2 mm of horizontal or downsloping ST segment depression, or a drop of systolic blood pressure > 20 mmHg during progressive exercise. VA other than sustained VT, including unifocal or multifocal ectopy, NSVT without hemodynamic stability, ventricular triplets and couplets were considered as nonterminating. Arrhythmias were tracked actively during the testing and registered by ECG tracings. All subjects were also evaluated by performing the 6 minutes walk test (6MWT) as a measure of submaximal exercise performance.

### 2.6 Statistical analysis

The results are expressed by classic descriptive parameters—mean and SD for parametric variables and median for variables that were not normally distributed. In order to apply parametric statistics, analysis of distribution was performed by the Kolmogorov‐Smirnov test, followed by power transformation of not normally distributed data. Categorical data are expressed as percentages. The unpaired *t* test was used to assess differences in key continuous variables between subjects who did and did not demonstrate NTVA during the test. The chi‐square test assessed differences in categorical data between these subgroups. Univariate and multivariate Cox regression analysis was used to assess the prognostic value of key CPET and Echo measures. For the multivariate regression, we used a forward conditional model with stepwise entry and removal criteria set at 0.05 and 0.10, respectively. Maximal iterations were set at 20. Kaplan‐Meier analysis was further used to assess the prognostic value of an exercise induced NTVA. The SPSS 22.0 (IBM, Armonk, New York) statistical software package was used for all analyses. All tests with a *P*‐value <.05 were considered statistically significant.

## 3 RESULTS

Of 319 subjects with HF enrolled, mean age 63.0 ± 9.9 years, 78% were male. LVEF of studied population was 36.0 ± 11.1% and 62% of them had ischemic etiology of HF. Of 319 subjects, 198 (62.1%) were diagnosed with HFrEF; 80 (25.2%) with HFmrEF, and 41 (12.9%) with HFpEF. There were no major cardiac events, implantable cardioverter defibrillator activations or deaths during testing. Of 319 subjects with HF 47 (15%) demonstrated NTVA during CPET, whereas one patient with HFrEF (0.3%) demonstrated sustained VT that indicated test termination and died 42 days after; this subject was excluded from further analysis. 31/166 (18.7%) patients with HFrEF, 12/80 (15.0%) patients with HFmrEF and 4/41 (9.8%) patients with HFpEF demonstrated a NTVA during CPET.

Clinical and echocardiographic data of patients with and without NTVA during CPET are shown in Table 1S.

Patients with NTVA had a lower LVEF, higher plasma NT‐pro‐BNP at rest and peak exercise, higher NYHA class and heart rate (HR) at rest, whereas 6MWT distance tended to be shorter but did not reach statistical significance. Age, body mass index and the distribution of males and females and the presence of CAD were similar between patients with and without NTVA, as well as HFrEF, HFmrEF, and HFpEF etiology (*P* > .05). There was no difference in beta blockers, statins, and angiotensin converting enzyme or angiotensin II receptor blocker usage in patients with and without NTVA, whereas patients with NTVA were more frequently prescribed a mineralocorticoid receptor blocker. Resting TAPSE was highly significantly lower and PASP higher in the subjects who exhibited NTVA (*P* < .001). Moreover, the TAPSE/PASP ratio, a measure of right ventricular‐pulmonary vasculature (RV‐PV) uncoupling,^18^ was lower in those with a NTVA during CPET (*P* < .001).

Subjects divided into groups according to presence or absence of the NTVA during CPET, showed a number of significant differences in CPET responses, as listed in Table 1. All patients reached metabolic criteria for maximal exercise test (RER >1.0); patients with NTVA exhibited higher RER. On average, subjects who exhibited NTVA had an unfavorable CPET response, such as lower peak values for HR, HRR‐1, peak VO_2_, and peak partial pressure of end‐tidal CO_2_ (P_ET_CO_2_) as well as a higher VE/VCO_2_ slope, and EOV prevalence.

**TABLE 1:** CPET parameters in patients with and without NTVA

	No arrhythmias (n = 271)	NTVA (n = 47)	*P*
Peak VO_2_, ml•min^−1^•kg^−1^ (mean ± SD)	14.8 ± 4.5	12.9 ± 4.3	.008
VE/VCO_2_ slope (mean ± SD)	33.9 ± 7.9	39.2 ± 9.7	<.001
Peak P_ET_CO_2_, mm Hg (mean ± SD)	33.9 ± 5.1	30.4 ± 4.8	<.001
EOV, n (%)	104 (38.4%)	34 (72.3%)	<.001
Peak HR, beats/min (mean ± SD)	127 ± 17	118 ± 14	.001
HRR, beats/min (mean ± SD)	17 ± 4	14 ± 4	<.001
Peak SAP, mm Hg (mean ± SD)	178 ± 14	174 ± 14	.09
RER (mean ± SD)	1.06 ± 0.1	1.12 ± 0.01	.001

Abbreviations: CPET, cardiopulmonary exercise testing; EOV, exercise oscillatory ventilation; HR, heart rate; HRR, heart rate recovery; NTVA, nonterminating ventricular arrhythmias; P_ET_CO_2_, end‐tidal partial pressure of carbon dioxide; RER, respiratory exchange ratio; SAP, systolic arterial pressure; VCO_2_, carbon dioxide output; VE, ventilation; VO_2_, oxygen consumption; WR, work rate (SD = SD).

The trend of differences in crucial clinical, echocardiographic and CPET variables between patients with and without NTVA was held in subgroups of patients with HFrEF, HFmrEF, and HFpEF together, as shown in Tables 2aS and 2bS, with the exception of EF in patients with HFmrEF and HFpEF, which was similar regardless of the occurrence or absence of NTVA during CPX. Systolic arterial pressure at rest and peak exercise did not differ between groups in any subgroup of EF (*P* > .05). For echocardiographic study, interobserver variability, assessed in a sample size of 20% of total population, was 3.5% for M‐mode and 2D echocardiography, and 4.7% for Doppler variables.

Seventy‐one patients died during the tracking period (25.8 ± 26.4 months), 17 for noncardiac, and 54 for cardiac reasons including the one subject who exhibited sustained VT during testing and was thus excluded from the analysis. There were 2 cardiac transplantations, 4 LVAD implantations, and 41 cardiac rehospitalizations during the tracking period. Data on cardiac events and noncardiac mortality in subgroups of patients with HFrEF, HFmrEF, and HFpEF during the follow‐up period are reported in Table 3S. NTVA was a significant predictor of mortality for cardiac reasons, as the primary outcome (Hazard ratio: 5.3; 95% confidence interval: 3.1‐9.2; *P* < .001), whereas it did not predict noncardiac mortality (*P* = .36). In a multivariate model including NTVA appearance, peak VO_2_, the VE/VCO_2_ slope and EOV, only NTVA, EOV and the VE/VCO_2_ slope were retained in the regression, as shown in Table 2. Multivariate Cox analysis demonstrating predictive value of NTVA for secondary outcome is shown in Table 4S.

**TABLE 2:** Cox analysis for key CPET variables in the prediction of primary outcome

	*χ* ^2^	Hazard ratio	95% CI	*P*
Univariate analysis				
NTVA	44.8	5.3	3.1‐9.2	<.001
Peak VO_2_	6.3	0.9	0.8‐1.0	.012
VE/VCO_2_ slope	32.5	1.1	1.0‐1.1	<.001
EOV	38.4	0.2	0.1‐0.3	<.001
Multivariate analysis				<.001
NTVA	44.8	3.1	1.7‐5.4	<.001
EOV	30.6	0.3	0.1‐0.6	<.001
VE/VCO_2_ slope	7.3	1.0	1.0‐1.1	.008
Peak VO_2_	0.8 (residual)			>.05
				

*Note:* Number of events = 53, Censored cases = 260, Censored cases before the earliest event = 5.

Abbreviations: CI, confidence interval; CPET, cardiopulmonary exercise testing; EOV, exercise oscillatory ventilation; NTVA, nonterminating ventricular arrhythmias; VCO_2_, carbon dioxide output; VE, ventilation; VO_2_, oxygen consumption.

**FIGURE 1:**
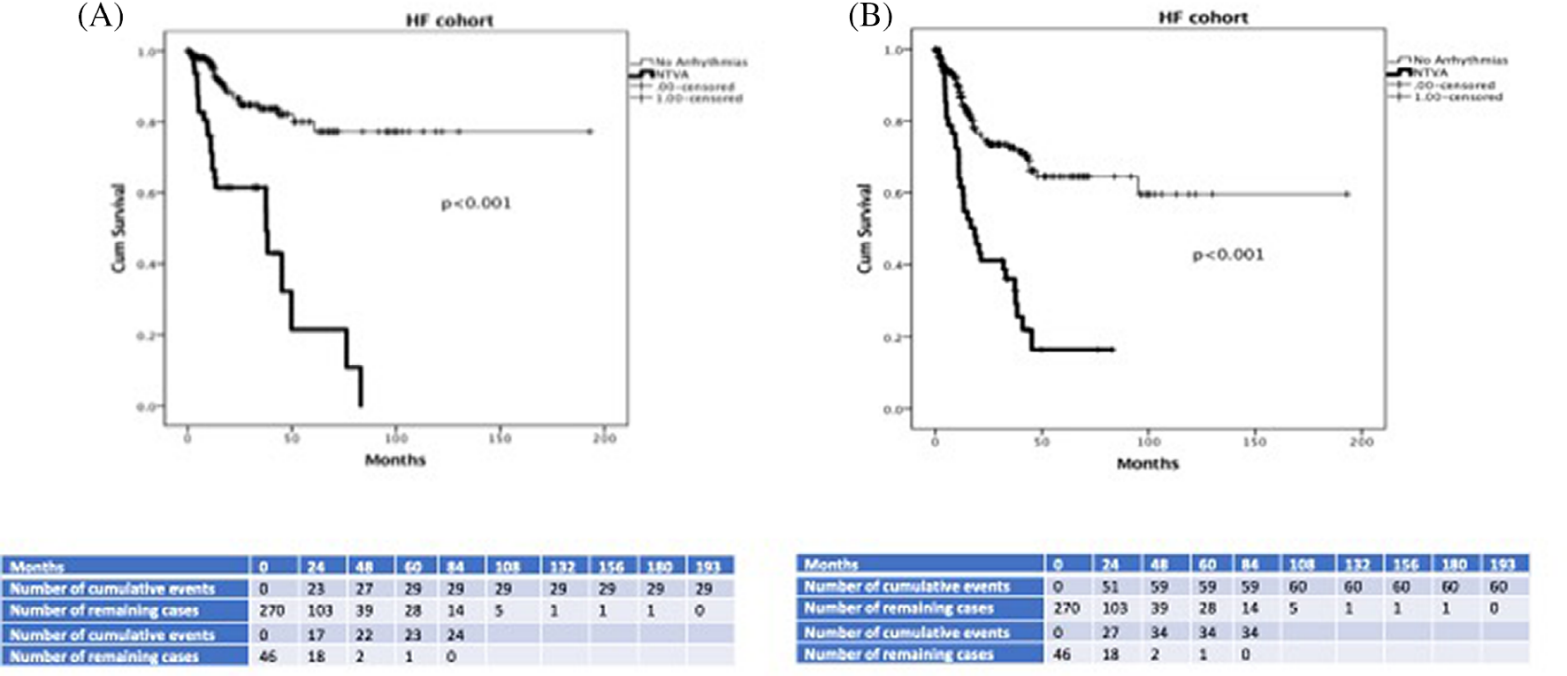
Kaplan‐Meier analysis of NTVA appearance during CPET in distinguishing between HF patients with and without primary (Figure 1A **)** and secondary outcome (Figure 1B) during 25.8 ± 26.4 months follow‐up period (*P* < .001). CPET, cardiopulmonary exercise testing; HF, heart failure; NTVA, nonterminating ventricular arrhythmias

On Kaplan Meier analysis, NTVA significantly distinguished patients with and without the primary (log‐rank Mantel‐Cox = 44.8, *P* < .001) and secondary outcome (log‐rank Mantel‐Cox = 37.4, *P* < .001) during the tracking period, as shown in Figure 1A,B. The same was shown for the patients enrolled in the following time periods: (a) 1994‐2004 (n = 187; Log Rank‐Mantel Cox = 33.5, 31.6, *P* < .001) and (b) 2005‐2015 (n = 130; Log Rank‐Mantel Cox = 7.6, 9.6, *P* < .01).

**FIGURE 2:**
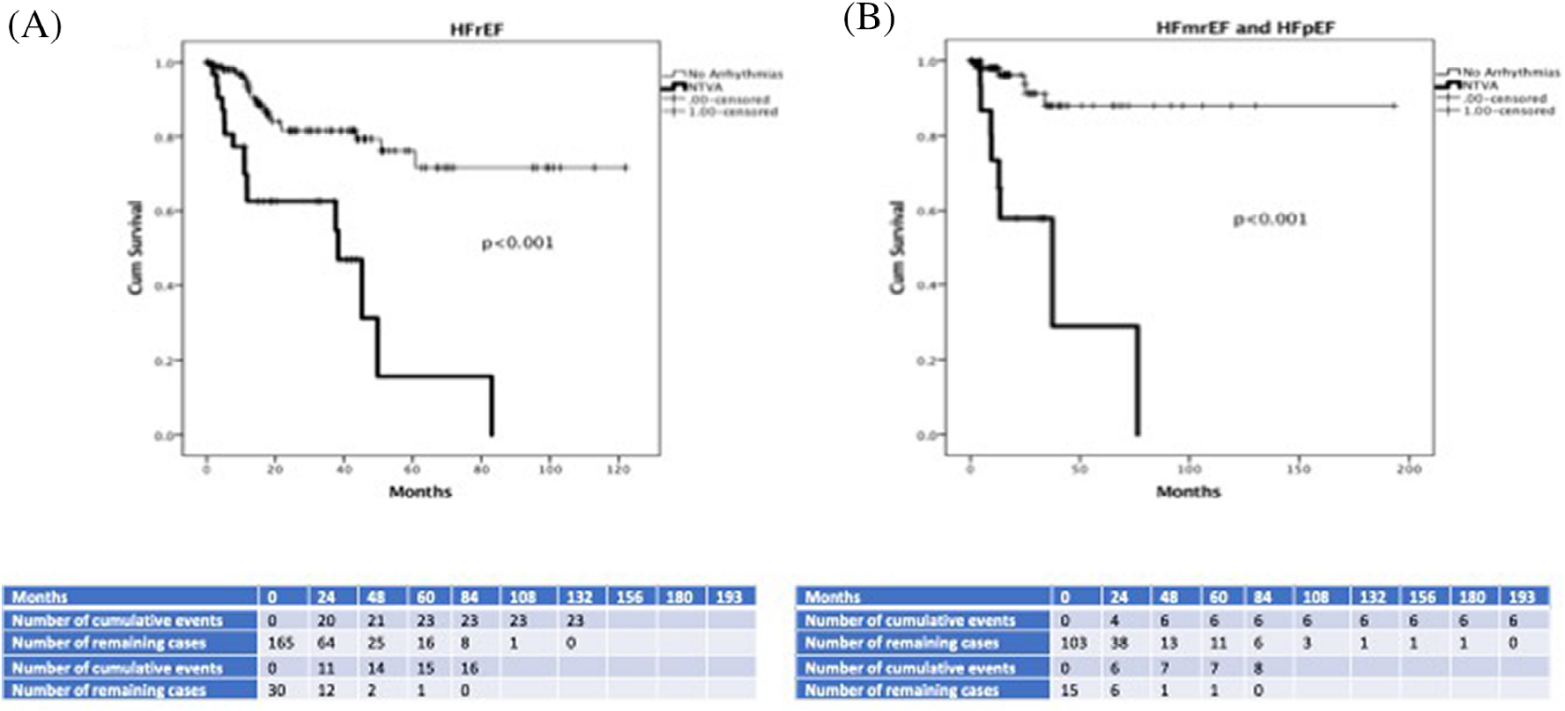
Kaplan‐Meier analysis of NTVA appearance during CPET in distinguishing between patients with and without primary outcome in HFrEF during 25.9 ± 24.3 months (Figure 2A **)** and HFmrEF/HFpEF during 25.6 ± 29.7 months follow‐up period (Figure 2B). CPET, cardiopulmonary exercise testing; HF, heart failure; HFrEF, HF with reduced EF; HFpEF, HF with preserved EF; HFmrEF, HF midrange EF; NTVA, nonterminating ventricular arrhythmias

**FIGURE 3:**
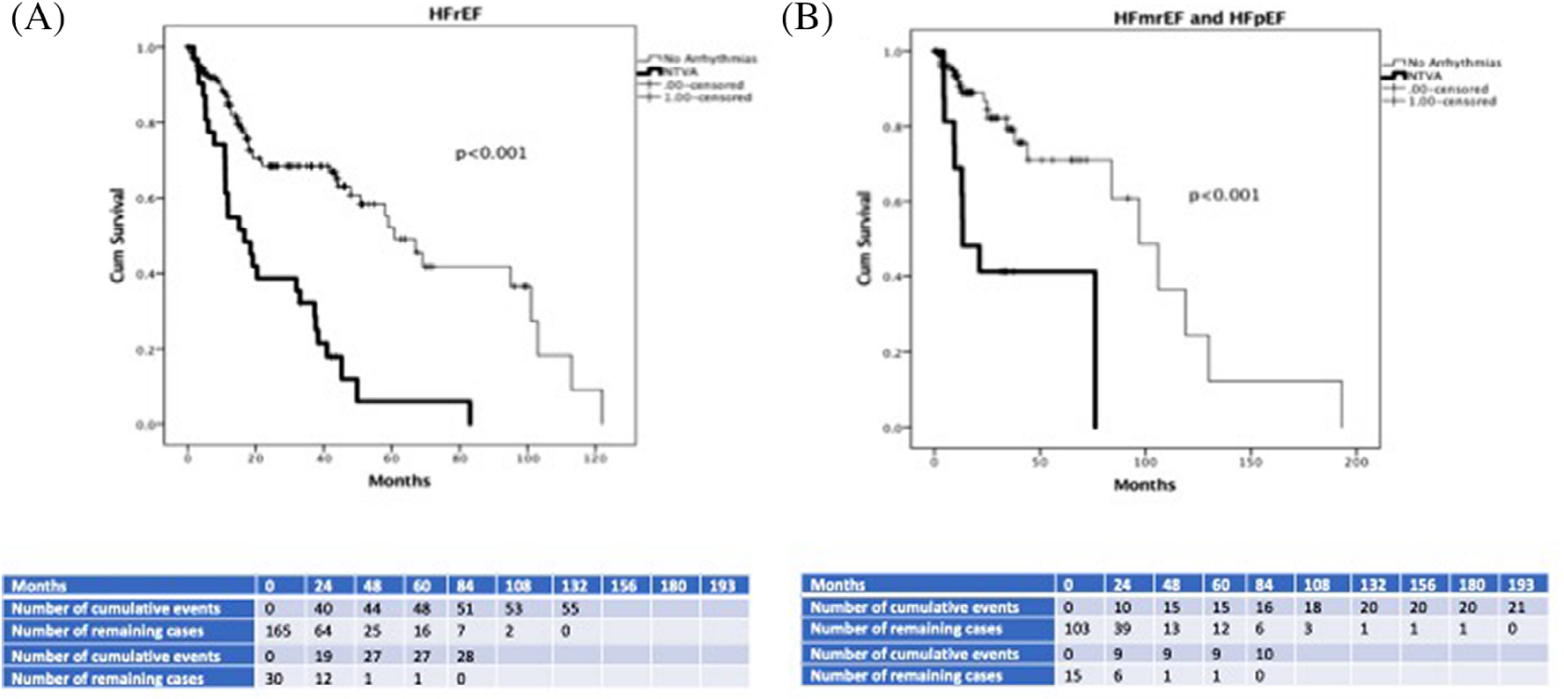
Kaplan‐Meier analysis of NTVA appearance during CPET in distinguishing between patients with and without secondary outcome in HFrEF during 25.9 ± 24.3 months (Figure 3A **)** and HFmrEF/HFpEF during 25.6 ± 29.7 months follow‐up period (Figure 3B **)**. CPET, cardiopulmonary exercise testing; HF, heart failure; HFrEF, HF with reduced EF; HFpEF, HF with preserved EF; HFmrEF, HF with midrange EF; NTVA, nonterminating ventricular arrhythmias

Kaplan Meier analysis showed that NTVA appearance during CPET significantly distinguished patients with and without primary and secondary outcome during the tracking period in subgroups of patients with HFrEF (log‐rank Mantel‐Cox = 23.6, 30.3, *P* < .001, respectively), and HFmrEF and HFpEF together (log‐rank Mantel‐Cox = 23.6, 15.7, *P* < .001, respectively), as shown in Figures 2A,B and 3A,B.

## 4 DISCUSSION

This is the first study addressing the potential for phenotyping HF severity and prediction of cardiovascular risk by looking at CPET variables in the presence of VA. The notable information is that the risk is remarkably increased even though subjects did not reach clinically recommended criteria to end the test. Subject with VA may exercise at maximum as suggested by RER at peak exercise. VA occurred in 15% of patients diagnosed with HF regardless of CAD prevalence, showing a strong predictive value for cardiac mortality and all‐cause mortality in combination with composite cardiac events, outperforming well‐established CPET parameters, such as peak VO_2_ and the VE/VCO_2_ slope. NTVA during CPET was related to higher rest and peak plasma NT‐pro‐BNP levels, lower LVEF, and a more unfavorable response of a host of CPET variables, including peak HR and HRR‐1. In the subjects with NTVA during CPET, indirectly measured pulmonary systolic pressure was increased, right ventricular systolic function depressed and the TAPSE/PASP ratio, a measure of RV‐PV uncoupling, low. Additionally, NTVA appearance during CPET occurred in a similar rate in all phenotypes, that is, HFrEF, HFmrEF, and HFpEF holding comparable prognostic power.

### 4.1 Pathophysiological insights into VA appearance during exercise in HF

It is well established that the VA rate in HF patients is associated with electrical conduction heterogeneity secondary to myocardial scar, ischemia or QT dispersion due to certain drugs, myocardial late potentials or re‐entry phenomena, and sympathetic system activation.^19^ Exercise may suppress cardiac arrhythmias detectable at rest by an overdrive suppression of the ectopic Purkinje pacemaker activity through sinus tachycardia favored by increased sympathetic tone and vagal withdrawal.^19^ On the other hand, increased sympathetic tone during exercise may induce ectopic impulse formation in the Purkinje tissue by increasing automaticity due to acceleration of the phase 4 of the action potential, provoking spontaneous discharge.^19^ Accordingly, in the present study, patients with NTVA achieved higher RER, suggesting the relation of higher metabolic achievements and VA expression. Ectopic ventricular beats are the most common cardiac arrhythmia during exercise, and they are usually associated with cardiac abnormalities, older age and obesity.^7^, ^19^ Furthermore, exercise‐related types of VA include catecholamine‐triggered polymorphic VT, but also right ventricular outflow tract VT associated usually with arrhythmogenic right ventricular dysplasia, suggesting the importance of the right ventricular pathology in generating VA.^19^

Actually, we do not have specific elements to dissect, which trigger mechanism may be predominant in our population. Nonetheless, no association was found between NTVA with older age, obesity, or increased CAD prevalence, suggesting that HF itself promotes some hemodynamic and metabolic derangements responsible for a lower oxygenation of myocardial cells. One explanation of this phenomenon may be the drop in cardiac output in association with the high catecholamine levels, followed by generalized vasodilation in exercising muscles, further affecting cardiac output.^1^, ^13^, ^19^, ^20^ This condition may lead to a reduction in coronary perfusion while HR is still elevated.

NTVA during CPET was associated with lower EF and RV to PC coupling, which is indicative for lower cardiac output during CPET. Accordingly, patients with NTVA demonstrated increased plasma BNP at rest and peak exercise which reflect higher myocardial stress and consequently lower contractility.^1^ However, in the group of patients with HFmrEF and HFpEF NTVA appearance during CPET was not related to a lower LV EF suggesting other implicated mechanisms.

A role would have been played by chronotropic incompetence,^21^ leading to insufficient cardiac output increase during exercise. In accordance with literature data, a lower HR at peak exercise in NTVA patients was observed regardless of beta receptor blockade. HRR‐1, a prognostic indicator in HF, was significantly lower as well. Overall, the constellation of these factors^21^, ^22^, ^23^ reflects the strong role of autonomic dysregulation which may be the basis of NTVA appearance, as well.

The fact that in patients with NTVA during CPET pulmonary vascular pressure was increased, systolic function of the right ventricle diminished, and the TAPSE/PASP ratio, a measure of RV‐PV uncoupling, decreased, may point toward a role of the right heart and pulmonary vasculature activity in generating arrhythmias.^24^ It seems reasonable to speculate that right ventricle and left ventricular cardiac output decrease, with a likely anticipation for the right ventricle. Namely, in the condition of right ventricular failure and increased PASP, the blood flow from the right heart to the left heart is usurped, leading to a reduction in cardiac output during exercise that could not be explained by the HR response per se.

It was shown previously that patients prescribed with nitrates show VA less likely.^5^, ^7^, ^25^ It is also known that exercise can induce cardiac arrhythmias under the conditions of diuretic and digitalis therapy.^7^, ^26^ Our results supported this finding, showing more NTVA during CPET in patients prescribed with mineralocorticoid receptor blockers. The explanation of this finding may be driven from electrolyte disturbances due to diuretic usage.^7^

Altogether, NTVA appearance during exercise in HF patients is apparently multifactorial, including lower oxygenation of the heart due to decreased cardiac output and usurped both the right and left ventricle, as well as endothelial dysfunction, metabolic derangements, autonomic dysregulation and therapeutic interventions.

### 4.2 Prognostic significance of NTVA during exercise testing in HF patients

Regardless of underlying mechanisms for VA appearance, an added novelty of the present study is the demonstrated prognostic significance of NTVA during CPET in HF patients. There is extensive literature demonstrating the prognostic value of abnormal responses during CPET in patients diagnosed with HF, such as low peak VO_2_, an elevated VE/VCO_2_ slope and EOV.^10^, ^27^ The present study revealed that NTVA appearance during CPET overperformed these already firmly established parameters indicating elevated risk for adverse events. Previous reports addressing the prognostic value of VA during and after exercise have been inconsistent.^7^, ^8^, ^9^ Some studies have shown that frequent or complex repetitive ventricular activity during exercise, and especially ventricular ectopy in the recovery period after exercise, heralds increased risk of death.^8^, ^9^, ^28^ Furthermore, exercise‐induced VA is an independent predictor of cardiovascular mortality and, in combination with resting premature ventricular contractions, carries the highest risk.^9^ Moreover, an origin of the ventricular ectopy was suggested as important in a prognostic sense, indicating that ectopy with a right bundle‐branch block morphology, common in patients with LV dysfunction, more likely predict adverse events than ectopy originating from the right ventricular outflow tract.^29^ The present study proposes the existence of a potential link between VA appearance and right heart function, both significantly determining prognosis, which is in accordance to previous studies which already demonstrated that right heart function is a crucial determinant of outcome in HF patients regardless of LV function or predominance of systolic or diastolic HF.^16^ It seems that the worse outcome in patients with HF is diminished right‐sided cardiac function and increased pulmonary pressure, with a real‐time decrease in left‐sided cardiac output, followed by arrhythmogenic presentation.

The finding that NTVA appearance during CPET demonstrated strong predictive value for cardiac events in the subgroups of patients with HFrEF, HFmrEF and HFpEF has to be stressed taking into consideration the poor availability of prognostic criteria for HFmrEF.^1^ A similar prognostic value of NTVA across HF subgroups suggests that there are some common pathophysiological mechanisms with similar prognostic implications.

Finally, considering the link of NTVA and worse prognosis, their occurrence should be a flywheel for rephrasing therapeutic approach, pharmacological or invasive, in order to prevent unwanted outcome.

Of note, in the present study, prognostic value of NTVA was not analyzed independently of EF and NT‐pro‐BNP, since the focus was to examine the overall power of CPET in HF risk stratification. Precise quantification of NTVA and determination of level of effort when NTVA appear may further improve prognostic power. Possible limitation of NTVA prognostic power may be presence of other conditions, such as respiratory diseases, sleep apnea, autonomic dysregulation, LV diastolic dysfunction or the use of different medications.^1^, ^5^ Although our subanalysis demonstrated similar results in the two time periods, from 1994 to 2004 and from 2005 to 2015, the long period of enrolment and variable follow‐up in this study could lead to a heterogeneity in the population, at least from the perspective of amelioration of medical therapies. Further, collection of data and studies with larger number of patients, with evaluation of exact mechanism of death, is needed to strengthen the conclusions on the prognostic value of NTVA in all subgroups of HF patients. Furthermore, validation of the findings of the present study from other independent cohorts is needed to confirm the clinical utility of our findings. Another limitation of this study is the lack of specific definition of pathophysiological mechanisms leading to NTVA and no in‐depth evaluation of structural cardiac muscle changes; however, we used strict echocardiographic protocol for noninvasive assessment rejecting data without good quality to minimize potential errors. Additionally, although NTVA during exercise can be easily registered and interpreted, technical improvement of ECG recordings is warranted. In our study, we used data derived from CPET, a comprehensive method for evaluation of HF patients, however, its broad applicability is lacking. Nonetheless, it is reasonable to speculate that our results may be transferable to standard exercise testing procedure and together with 24 hours Holter monitoring may further contribute to more efficient risk stratification in HF patients.

In conclusion, exercise‐induced arrhythmias not reaching criteria for test termination seem nonetheless indicative of an advanced HF severity phenotype, and worse prognosis, independently of HF subtype. Marked abnormalities in CPET‐derived variables drive the outcome prediction. Among the many potential causes, our data suggest a role of unfavorable combination of RV‐PV uncoupling and autonomic dysregulation leading to reduced cardiac output during exercise.

### 4.3 Clinical perspectives: Competencies in medical knowledge and translational outlook

In HF of any left ventricular ejection fraction, mortality remains high and multiparametric assessment of risk is of basic importance. The search for best phenotyping at different stages and with multiparametric approach is challenging and matter of intense investigation. Ventricular arrhythmias triggered by exercise testing not meeting test‐termination criteria appear an underestimated manifestation that increases in rate of presentation according to disease severity and portends an unfavorable prognosis in HF, regardless of left ventricular EF.

## CONFLICT OF INTERESTS

The authors have no conflicts of interest to disclose.

## Supporting information

Table 1S Clinical and echocardiographic characteristics of study patients with and without NTVA during CPET

Table 2S Table 2A Clinical, echocardiographic and CPET characteristics of patients diagnosed with HFrEF, with and without NTVA
Table 2bS. Clinical, echocardiographic and CPET characteristics of patients diagnosed with HFmrEF and HFpEF, with and without NTVA

Table 3S Follow up of patients with HFrEF, HFmrEF and HFpEF

**Table 4S** Cox analysis for key CPET variables in the prediction of secondary outcome
