# Supplementary material for: Ventricular arrhythmias not meeting criteria for terminating cardiopulmonary exercise testing stratify prognosis and disease severity in heart failure of preserved, midrange, and reduced ejection fraction
Source: Clin Cardiol. 2020 Apr 9;43(7):698–705. doi: 10.1002/clc.23367 (PMC7368295; doi:10.1002/clc.23367)
Supplement: Supplementary file 1 — Table 1S Clinical and echocardiographic characteristics of study patients with and without NTVA during CPET [file CLC-43-698-s001.doc]

**Table 1S.** **Clinical and echocardiographic characteristics of study patients with and without NTVA during CPET**

|  | **No Arrhythmias (n=271)** | | | | **NTVA (n=47)** | | | | **P Value** |
| --- | --- | --- | --- | --- | --- | --- | --- | --- | --- |
| **Age, y (mean ± SD)** | 62.8 | ± | | 9.8 | 64.3 | ± | 10.8 | | >0.05 |
| **Female sex, n (%)** | 61 (22.5%) | | | | 7 (14.9%) | | | | >0.05 |
| **BMI, kg/m^2^ (mean ± SD)** | 26.7 | ± | | 4.5 | 26.4 | ± | 3.9 | | >0.05 |
| **CAD, n (%)** | 169 (62.4%) | | | | 30 (63.8%) | | | | >0.05 |
| **NYHA class, n (%)** |  |  | |  |  |  |  | |  |
| **I** | 45 (16.7%) | | | | 1 (2.1%) | | | | 0.009 |
| **II** | 148 (54.6%) | | | | 20 (42.6%) | | | | >0.05 |
| **III** | 70 (25.8%) | | | | 23 (48.9%) | | | | 0.003 |
| **IV** | 5 (1.9%) | | | | 3 (6.4%) | | | | >0.05 |
| **NT-pro-BNP rest, pg/ml (median ± IQR)** | 874.0 | ± | 741.0 | | 1470.0 | ± | 1127.0 | | <0.001 |
| **NT-pro-BNP peak, pg/ml (median ± IQR)** | 920.0 | ± | 779.0 | | 1680.0 | ± | 1129.0 | | <0.001 |
| **EF, % (mean ± SD)** | 36.7 | ± | 10.9 | | 32.6 | ± | 11.1 | | 0.02 |
| **PASP, mm Hg (mean ± SD)** | 38.2 | ± | 10.8 | | 45.7 | ± | 11.7 | | <0.001 |
| **TAPSE, mm (mean ± SD)** | 18.2 | ± | 3.0 | | 15.8 | ± | 2.9 | | <0.001 |
| **TAPSE/PASP, mm/mm Hg (mean ± SD)** | 0.53 | ± | 0.19 | | 0.37 | ± | 0.13 | | <0.001 |
| **SAP, mm Hg (mean ± SD)** | 122 | ± | 9 | | 118 | ± | 13 | | 0.012 |
| **HR, beats/min (mean ± SD)** | 73 | ± | 8 | | 78 | ± | 10 | | <0.001 |
| **HFrEF, n (%)** | 166 (61.3%) | | | | 31 (66.0%) | | | | >0.05 |
| **HFmrEF, n (%)** | 68 (25.1%) | | | | 12 (25.5%) | | | | >0.05 |
| **HFpEF, n (%)** | 37 (13.7%) | | | | 4 (8.5%) | | | | >0.05 |
| **6MWT, m (mean ± SD)** | 359.2 | ± | | 91.4 | 331.8 | ± | | 92.1 | 0.059 |
| **Medications** |  |  | |  |  |  | |  |  |
| **Beta blockers, n (%)** | 174 (64.2%) | | | | 34 (72.3%) | | | | >0.05 |
| **ACE-inhibitor or ARB, n (%)** | 216 (79.7%) | | | | 43 (91.5%) | | | | 0.055 |
| **Aldosterone antagonist, n (%)** | 125 (46.1%) | | | | 31 (66.0%) | | | | 0.019 |
| **Statins, n (%)** | 156 (57.6%) | | | | 25 (53.2%) | | | | >0.05 |

ACE = angiotensin converting enzyme, ARB = angiotensin II receptor blocker, BMI = body mass index, NT-pro-BNP = N-terminal pro-brain natriuretic peptide, CAD = coronary artery disease, EF = ejection fraction, HFpEF = heart failure with preserved ejection fraction, HFmrEF = heart failure with middle-ranged ejection fraction, HFrEF = heart failure with reduced ejection fraction, HR = heart rate, NTVA = non terminating ventricular arrhythmias, NYHA = New York Heart Association, SAP = systolic arterial pressure, PASP = systolic pulmonary artery pressure, TAPSE = tricuspid annular plain systolic excursion, 6MWT = six minute walk test (SD = standard deviation, IQR = interquartile range)
