# Supplementary material for: Ventricular arrhythmias not meeting criteria for terminating cardiopulmonary exercise testing stratify prognosis and disease severity in heart failure of preserved, midrange, and reduced ejection fraction
Source: Clin Cardiol. 2020 Apr 9;43(7):698–705. doi: 10.1002/clc.23367 (PMC7368295; doi:10.1002/clc.23367)
Supplement: Supplementary file 2 — Table 2S Table 2A Clinical, echocardiographic and CPET characteristics of patients diagnosed with HFrEF, with and without NTVA Table 2bS. Clinical, echocardiographic and CPET characteristics of patients diagnosed with HFmrEF and HFpEF, with and without NTVA [file CLC-43-698-s002.doc]

**Table 2bS.** **Clinical, echocardiographic and CPET characteristics of patients diagnosed with HFmrEF and HFpEF, with and without NTVA**

|  | **No Arrhythmias (n=105)** | | | **NTVA (n=16)** | | | **P Value** |
| --- | --- | --- | --- | --- | --- | --- | --- |
| **NT-pro-BNP rest, pg/ml (median ± SD)** | 918.4 | ± | 640.0 | 1783.9 | ± | 767.8 | <0.001 |
| **NT-pro-BNP peak, pg/ml (median ± SD)** | 994.6 | ± | 687.0 | 1935.5 | ± | 833.9 | <0.001 |
| **6MWT, m (mean ± SD)** | 373.4 | ± | 97.9 | 320.3 | ± | 93.1 | 0.051 |
| **EF, % (mean ± SD)** | 47.6 | ± | 7.4 | 45.5 | ± | 6.3 | >0.05 |
| **PASP, mm Hg (mean ± SD)** | 36.3 | ± | 11.4 | 47.0 | ± | 12.4 | 0.001 |
| **TAPSE, mm (mean ± SD)** | 19.0 | ± | 3.1 | 15.9 | ± | 3.5 | <0.001 |
| **TAPSE/PASP, mm/mm Hg (mean ± SD)** | 0.58 | ± | 0.21 | 0.37 | ± | 0.16 | <0.001 |
| **Peak VO_2_, ml⚫min^-1^⚫kg^-1^ (mean ± SD)** | 15.5 | ± | 4.8 | 12.2 | ± | 4.4 | 0.014 |
| **VE/VCO_2_ slope (mean ± SD)** | 32.6 | ± | 7.5 | 38.2 | ± | 6.5 | 0.007 |
| **Peak P_ET_CO_2_, mm Hg (mean ± SD)** | 34.9 | ± | 5.2 | 30.0 | ± | 4.1 | 0.001 |
| **EOV, n (%)** | 26 (24.8%) | | | 12 (75.0%) | | | 0.001 |
| **HR rest, beats/min (mean ± SD)** | 72 | ± | 8 | 79 | ± | 11 | 0.025 |
| **Peak HR, beats/min (mean ± SD)** | 128 | ± | 18 | 117 | ± | 14 | 0.004 |
| **HRR, beats/min (mean ± SD)** | 18 | ± | 4 | 15 | ± | 3 | 0.003 |

NT-pro-BNP = N-terminal pro-brain natriuretic peptide, EF = ejection fraction, EOV = exercise oscilatory ventilation, HFrEF = heart failure with reduced ejection fraction, HR = heart rate, HRR = heart rate recovery, 6MWT = six minute walk test, NTVA = non-terminating ventricular arrhythmias, PASP = systolic pulmonary artery pressure, P_ET_CO_2_ – end-tidal partial pressure of carbon-dioxide, TAPSE = tricuspid annular plain systolic excursion, VCO_2_ = carbon dioxide output, VE = ventilation, VO_2_ = oxygen consumption, WR = work rate.
