# Supplementary material for: Ventricular arrhythmias not meeting criteria for terminating cardiopulmonary exercise testing stratify prognosis and disease severity in heart failure of preserved, midrange, and reduced ejection fraction
Source: Clin Cardiol. 2020 Apr 9;43(7):698–705. doi: 10.1002/clc.23367 (PMC7368295; doi:10.1002/clc.23367)
Supplement: Supplementary file 3 — Table 3S Follow up of patients with HFrEF, HFmrEF and HFpEF [file CLC-43-698-s003.docx]

**Table 3S. Follow up of patients with HFrEF, HFmrEF and HFpEF**

|  |  |  |  |
| --- | --- | --- | --- |
|  | **HFrEF**  **n=198** | **HFmrEF/HFpEF**  **n=121** | **Total**  **n=319** |
| **Cardiac death** | 40 (20.2) | 14 (11.6) | 54 (16.9) |
| **Other cause death** | 10 (5.1) | 7 (5.7) | 17 (5.3) |
| **Cardiac transplantation** | 1 (0.5) | 1 (0.8) | 2 (0.6) |
| **LVAD implantation** | 3 (1.5) | 1 (0.8) | 4 (1.3) |
| **Rehospitalisation for cardiac reasons** | 31 (15.7) | 10 (8.3) | 41 (12.9) |

LVAD = Left ventricular assist device, HFrEF = heart failure with reduced ejection fraction, HFmrEF = heart failure with middle range ejection fraction, HFpEF = heart failure with preserved ejection fraction
