# Supplementary material for: Ventricular arrhythmias not meeting criteria for terminating cardiopulmonary exercise testing stratify prognosis and disease severity in heart failure of preserved, midrange, and reduced ejection fraction
Source: Clin Cardiol. 2020 Apr 9;43(7):698–705. doi: 10.1002/clc.23367 (PMC7368295; doi:10.1002/clc.23367)
Supplement: Supplementary file 4 — Table 4S Cox analysis for key CPET variables in the prediction of secondary outcome [file CLC-43-698-s004.doc]

**Table 4S.** Cox analysis for key CPET variables in the prediction of secondary outcome

|  | **χ^2^** | **Hazard Ratio** | | | **95% CI** | **P Value** |  | | |
| --- | --- | --- | --- | --- | --- | --- | --- | --- | --- |
|  | **Univariate analysis** | | | | | |  | | |
| **NTVA** | 45.8 | 3.7 | | 2.4-5.5 | | <0.001 |  | | |
| **Peak VO_2_** | 30.7 | 0.9 | | 0.8-0.9 | | <0.001 |  | | |
| **VE/VCO_2_ slope** | 47.2 | 1.1 | | 1.0-1.1 | | <0.001 |  | | |
| **EOV** | 61.7 | 0.2 | | 0.1-0.3 | | <0.001 |  | | |
|  | **Multivariate analysis** | | | | | |  |  | <0.001 |
| **NTVA** | 38.8 | 2.0 | 1.3-3.1 | | | 0.001 |  | | |
| **EOV** | 61.7 | 0.3 | 0.2-0.5 | | | <0.001 |  | | |
| **VE/VCO_2_ slope** | 5.9 | 1.0 | 1.0-1.1 | | | 0.021 |  | | |
| **Peak VO_2_** | 21.1 | 0.9 | 0.8-1.0 | | | 0.001 |  | | |
|  |  |  |  | | |  |  | | |

CI – confidence interval, EOV = exercise oscilatory ventilation, NTVA = non-terminating ventricular arrhythmias, VCO_2_ = carbon dioxide output, VE = ventilation, VO_2_ = oxygen consumption

Number of events = 115, Censored cases = 202, Censored cases before the earliest event = 1.
